# The cost of relapse and the predictors of relapse in the treatment of schizophrenia

**DOI:** 10.1186/1471-244X-10-2

**Published:** 2010-01-07

**Authors:** Haya Ascher-Svanum, Baojin Zhu, Douglas E Faries, David Salkever, Eric P Slade, Xiaomei Peng, Robert R Conley

**Affiliations:** 1 US Outcomes Research, Eli Lilly and Company, Lilly Corporate Center, Indianapolis, IN 46285, USA; 2 US Statistics, Lilly USA, LLC, Lilly Corporate Center, Indianapolis, IN 46285, USA; 3 Department of Public Policy, University of Maryland, Baltimore County, 1000 Hilltop Circle, Baltimore, MD 21250, USA; 4 University of Maryland School of Medicine, 655 West Baltimore Street, Baltimore, MD 21201, USA; 5 VA VISN 5 Mental Illness Research, Education, and Clinical Center, US Department of Veterans Affairs, 10 North Greene Street, Baltimore, MD 21201, USA; 6 US Medical Division, Lilly USA, LLC, Lilly Corporate Center, Indianapolis, IN 46285, USA

## Abstract

**Background:**

To assess the direct cost of relapse and the predictors of relapse during the treatment of patients with schizophrenia in the United States.

**Methods:**

Data were drawn from a prospective, observational, noninterventional study of schizophrenia in the United States (US-SCAP) conducted between 7/1997 and 9/2003. Patients with and without relapse in the prior 6 months were compared on total direct mental health costs and cost components in the following year using propensity score matching method. Baseline predictors of subsequent relapse were also assessed.

**Results:**

Of 1,557 participants with eligible data, 310 (20%) relapsed during the 6 months prior to the 1-year study period. Costs for patients with prior relapse were about 3 times the costs for patients without prior relapse. Relapse was associated with higher costs for inpatient services as well as for outpatient services and medication. Patients with prior relapse were younger and had onset of illness at earlier ages, poorer medication adherence, more severe symptoms, a higher prevalence of substance use disorder, and worse functional status. Inpatient costs for patients with a relapse during both the prior 6 months and the follow-up year were 5 times the costs for patients with relapse during the follow-up year only. Prior relapse was a robust predictor of subsequent relapse, above and beyond information about patients' functioning and symptom levels.

**Conclusions:**

Despite the historical decline in utilization of psychiatric inpatient services, relapse remains an important predictor of subsequent relapse and treatment costs for persons with schizophrenia.

## Background

Schizophrenia is a severe and chronic mental illness characterized by recurring relapses that may require inpatient hospitalization. Costs associated with treatment received consequent to relapse may account for the largest share of treatment costs in schizophrenia [1-4], which is one of the most expensive to treat psychiatric conditions [5]. Socio-demographic and clinical factors associated with relapse have been examined in previous research studies [2-4,6-9]. However, except for results from 1 published study [1], information about potential predictors of relapse and its associated treatment costs in the United Stated are scarce.

Information about the cost of relapse in schizophrenia and the predictors of relapse is of interest to clinicians, payers, and other health care decision makers. Intensive outpatient service interventions, such as assertive community treatment, partial hospitalization programs, and programs for persons with co-occurring addictive disorders, which are designed for persons at risk of acute relapse, could help prevent or minimize relapses and attendant health care costs. However, intensive outpatient interventions cost too much to be offered to all patients with schizophrenia who might benefit from them. As a result, accurate prediction of risk of relapse is critical to identifying persons who may need these intensive outpatient interventions.

In essentially the only study of the costs of relapse for persons treated for schizophrenia in the United States, Weiden and Olfson estimated that, on a national level, almost $2 billion is spent annually for hospital readmissions of patients with schizophrenia [1]. That study, though based on a national sample, was based on a cross-sectional database that contained limited information about illness severity and clinical outcomes over time. The data used in the present study were from a longitudinal observational study of persons treated for schizophrenia in usual-care settings in the United States. The purpose of the study was to estimate the direct annual mental health costs of relapse and its cost components, to identify predictors of relapse, and to clarify the role of recent, prior relapse on subsequent costs. It was hypothesized that patients with prior relapse will incur significantly higher total direct mental health cost in the following year than patients without prior relapse and that in addition to higher inpatient hospitalization cost they will incur significantly higher cost of outpatient services. We also hypothesized that patients with both prior and subsequent relapse will be the costliest and that prior relapse will be a significant predictor of subsequent relapse along with other distinct patient characteristics such as substance use and poor medication adherence.

## Methods

### Data source

Data were used from the US Schizophrenia Care and Assessment Program (US-SCAP), a large (N = 2,327) 3-year prospective, observational, noninterventional study of schizophrenia treatment in usual-care settings in the United States conducted between July 1997 and September 2003. Participants were recruited from diverse geographic areas, including the Northeast, Southwest, Mid-Atlantic, and West. The 6 participating regional sites represented large systems of care, including community mental health centers, university health care systems, community and state hospitals, and the Department of Veterans Affairs Health Services. Institutional Review Board approval was obtained, and informed consent was received from all participants.

Participants were ages 18 or older and had been diagnosed with schizophrenia, schizoaffective, or schizophreniform disorder based on *Diagnostic and Statistical Manual, Version 4 *criteria. Participants were excluded if they were unable to provide informed consent or had participated in a clinical drug trial within 30 days prior to enrollment. Approximately 400 patients enrolled at each of the 6 study sites. Enrollment was not contingent upon participants having been treated with any medication and was independent of concurrent psychiatric or medical conditions, use of concomitant medications, or substance use. Patients could stay on medications received prior to enrollment, and decisions about medication changes, if any, were made by the physicians and their patients. Further details about US-SCAP have been reported elsewhere [10,11].

### Analytical sample

Of 2,327 patients in the US-SCAP, 1,817 (78%) completed a 1-year follow-up interview. Of these 1,817 patients, the present analysis included only participants for whom complete mental health resource utilization data were available for an entire year (N = 1,557 or 85.7%). If more than 1 year of complete resource use information was available for a given patient, data from the earliest year were used. The first year of patients' participation in the study was often the study year.

In addition to comparing patients with and without prior relapse on baseline characteristics and on mental health costs, the impact of prior relapse on subsequent relapse (within the following year) was assessed. This resulted in 4 mutually exclusive groups: 1) patients who relapsed during both time periods (prior Relapse and subsequent Relapse, designated "RR"); 2) patients with No prior relapse but with subsequent Relapse (designated "NR"); 3) patients with prior Relapse but with No subsequent relapse (designated "RN"); and 4) patients who did not relapse during either time period (No prior relapse and No subsequent relapse, designated "NN").

### Measures

Relapse was defined as having any of the following: psychiatric hospitalization, use of emergency services, use of a crisis bed, or a suicide attempt. These relapse parameters, with the exception of suicide attempt, were based on information systematically abstracted from patients' medical records every 6 months, using an abstraction form developed for the study. Suicide attempts, for the previous 1-month period, were reported by the patients on the SCAP-Health Questionnaire (SCAP-HQ), a validated measure developed for the study [12].

Standard psychiatric measures were used to assess participant sociodemographic, clinical, and functional status at baseline. A structured interview was used to identify sociodemographic characteristics. Level of symptom severity was assessed annually with the Positive and Negative Syndrome Scale (PANSS) [13] and the Montgomery-Åsberg Depression Rating Scale (MADRS) [14]. Levels of functioning in various domains were assessed with the SCAP-HQ, which provided information on suicide attempts, violent behaviors, medication adherence, drug and alcohol use for the previous month, and arrests in the previous 6 months. Mental and physical levels of functioning were assessed with the 12-Item Short Form Health Survey (SF-12) [15].

Patient-reported medication adherence was assessed with SCAP-HQ on a 5-point scale. Participants who reported they "never missed" taking their medication or "missed only a couple of times but basically took all medicine" were considered adherent, whereas all others ("took at least half," "took less than half," or "stopped taking medication") were considered nonadherent. In addition to patient-reported adherence, medication adherence in the 6 months before the study year was measured by the Medication Possession Ratio (MPR) [2,6]. Using prescription information in patient medical records, the MPR was calculated as the proportion of days with any antipsychotic medication. An MPR value of at least .80 is considered being adherent [6]. Prior research found high correspondence between antipsychotic prescription and their pharmacy fill in this population [4], and the prescription-based MPR used in this analysis has previously provided results highly consistent with research using pharmacy fill-based MPR [10].

### Resource utilization and cost

Mental health resource utilization information for each participant was abstracted at baseline and every 6 months thereafter by trained examiners who used a medical record abstraction form developed for this study. At these time points, participants were also queried about treatment received outside their usual health care site, and study personnel obtained medical records from these treatment centers as needed. Total 1-year direct mental health costs included the following cost components: costs of medications (antipsychotics, other psychotropics, such as mood stabilizers, anticholinergics, antidepressants, antianxiety, and sleep agents), psychiatric hospitalizations, day treatment, emergency services, psychosocial group therapy, medication management, individual therapy, and ACT/case management. Consistent with prior antipsychotic drug cost research [16,17], the costs of atypical antipsychotic medications were based on average wholesale prices discounted by 15%, reflecting the customary discount level in the United States. Costs of psychiatric hospitalization were based on daily per diem costs at each site. To help address variations in resource utilization types, durations, and costs across study sites, the costs of mental health services other than psychiatric hospitalizations, were based on their relative value units developed from resource utilization and cost data available from the management information systems at each site [18,19]. Direct cost data were not available for the 6-month pre-study period, but data on relapse, including number of psychiatric hospitalizations and length of stay (LOS) were available.

### Statistical analysis

Initial statistical group comparisons assessed patients who relapsed during the prior 6 months compared with patients who did not (RR and RN versus NR and NN). Following this, pairwise comparisons among the 4 groups based on prior and subsequent relapse status (NN, NR, RR, and RN) were conducted. Group comparisons were performed using *t *tests for continuous variables and Mantel-Haenszel χ^2 ^tests for categorical variables. Average total direct mental health costs and cost components were assessed during the study year and were compared between patients who relapsed (in the 6 months preceding the 1-year follow-up) and those who did not using propensity score adjusted bootstrap resampling. Propensity score stratification [20] was used to adjust for potential confounding factors not attributable to relapse status. A priori covariates for calculating the logit score with this method were age; gender; race/ethnicity; illness duration; insurance status; a diagnosis of a schizoaffective disorder, comorbid substance use, personality disorder, or mental retardation; enrollment site; a binary indicator for psychiatric hospitalization at the time of enrollment into the US-SCAP study; and time elapsed between US-SCAP enrollment and the start date of each patient's study year. As a sensitivity analysis, the a priori propensity score model was modified to include all baseline covariates for which statistically significant group imbalance was found. The bootstrap resampling approach (1,000 iterations) was used to provide a nonparametric approach due to the skewness of the cost data.

To determine predictors of relapse during the 1-year study period, a stepwise logistic regression analyses was conducted for (1) all patients, (2) patients with prior relapse, and (3) patients without prior relapse.

## Results

### Patients with versus without prior relapse

Of 1,557 participants eligible for analyses, 310 (20%) relapsed in the 6 months prior to the study period, and 1,247 (80%) did not. As shown in Additional file 1, patients with prior relapse were significantly younger, with earlier age at illness onset, more severe schizophrenia symptoms and depressive symptoms, higher rates of psychiatric hospitalization in the year prior to enrollment in the study, substance use disorder, arrests, and victimization by others. They also had significantly poorer levels of mental health and were less likely to be adherent with medication (per self-report and MPR). Of the 310 patients with prior relapse, 281 (91%) had a psychiatric hospitalization, 41 (13%) used emergency services or crisis beds, and 20 (6%) reported suicide attempts (numbers exceed 100% because some patients met more than 1 relapse criterion). Most patients (258 of 310, or 83%) met 1 of these 4 criteria for relapse; 31 (10%) met 2; 21 (7%) met 3; and no participant met all 4. Only 1% of the patients (22 of 1557) were inpatients at the start of their 1-year study period.

Compared to patients who did not experience prior relapse, patients with prior relapse incurred significantly higher total annual direct mental health care costs during the 1-year study period, which were nearly 3 times higher for the relapsed ($33,187 ± $47,616) compared with those who did not ($11,771 ± $10,611, p < .01). Although the relapsed patients had significantly higher psychiatric hospitalization and emergency services costs, they also incurred significantly higher costs for medications and various outpatient services, including medication management, day treatment, individual therapy, and ACT/case management. Results were essentially unchanged when the a priori propensity score model was modified to include baseline covariates for which statistically significant group difference was found.

Furthermore, to help assess whether knowledge about previous relapse improves the ability to predict subsequent treatment costs over and above potential associations with patients' current level of functioning and symptomatology, we have conducted a sensitivity analysis. This analysis compared the total cost and cost components between patients with versus without relapse while adjusting for clinical and functional status as measured by the PANSS, MADRS, and SF12 (physical component score and mental component score) using propensity score estimation. Results of this sensitivity analysis were essentially the same, except that the original significant group differences on medication cost (with significantly higher medication cost for patients with prior relapse) became statistically non-significant. Findings support, therefore, that knowledge about previous relapse improves the ability to predict subsequent treatment costs above and beyond information about patients' functioning and symptom levels.

### Comparisons between groups by prior and subsequent relapse status

Among the 1,557 participants with eligible data, 1,078 (69%) did not relapse in the prior 6 months or during the subsequent 1-year study period (NN group), 157 (10%) experienced relapse during both periods (RR group), 169 participants (11%) did not have a prior relapse but relapsed during the 1-year study period (NR group), and the remaining 153 (10%) experienced prior relapse but did not relapse during the 1-year study period (RN group). These findings indicate that among the non-relapsed in the 1-year follow-up period, 87.6% (1078 of 1231) were correctly identified as non-relapsed based on their prior 6-month status (relapsed or not). This high specificity level was accompanied by moderate sensitivity (48.2%), high negative predictive value (86.4%), moderate positive predictive value (50.6%), and a high overall accuracy level (79.3%).

As shown in Additional file 2, significant differences were observed between these 4 groups on baseline characteristics and cost parameters. Compared to patients without prior relapse who relapsed in the subsequent year (NR), the patients with both prior and subsequent relapse (RR) were significantly younger, had a psychiatric hospitalization in the year prior to study enrollment, had more severe symptoms on the PANSS and MADRS, had poorer physical health functioning, and were more likely to be nonadherent per self-report and per medication records (MPR). Compared to the NR group, the group without prior or subsequent relapse (NN) was older, less likely to have comorbid substance-use disorder, had a psychiatric hospitalization in the year prior to study enrollment, had better mental and physical health functioning, and had less severe depressive symptoms. Compared to the NR group, patients with prior relapse but without subsequent relapse (RN) were younger, less likely to have health insurance, had a higher hospitalization rate in the year prior to study enrollment, and had better physical health functioning. Patients without prior or subsequent relapse (NN group) differed from those with both prior and subsequent relapse (RR group) on baseline variables associated with prior relapse, as noted earlier.

The 4 patient groups were also compared on total cost and cost components for the subsequent year (Additional file 2). As expected, the RR group was the costliest and was about 5 times more costly than the group who did not relapse (NN). Interestingly, the RR group was 2.4 times more costly than the NR group, although both groups relapsed during the 1-year study period, highlighting the impact of prior relapse on the total cost. In addition, the cost for the RN group was 1.5 times that of the NN group, demonstrating again the economic impact of prior relapse even when no subsequent relapse took place. Costs were driven primarily by psychiatric hospitalization and antipsychotic medications; the mean hospitalization cost for the RR group was almost 5 times that for the NR group ($38,104 vs. $7,786, p < .001). To better understand the drivers of the differences between the NR and RR groups on hospitalization costs during the 1-year study period, this analysis further compared them on hospitalization parameters. The RR group was found to have a significantly higher average LOS per psychiatric admission compared to the NR group (51.24 ± 101.41 vs. 9.84 ± 20.94 days, p < .001) and significantly more psychiatric hospitalizations (1.46 ± 1.22 vs. 0.99 ± 0.84, p < .001).

### Predictors of relapse

The predictors of relapse in the 1-year study for all patients and by prior relapse status are presented in Additional file 3. Overall (Additional file 3A), the predictors of subsequent relapse included presence of prior relapse, having health insurance, being medication nonadherent, younger at illness onset, and poorer functioning level. Among patients with prior relapse (RN vs. RR groups, Additional file 3B), the predictors were more severe schizophrenia symptoms per PANSS and a higher number of psychiatric hospital admissions in the prior year. Among patients without prior relapse (NN vs. NR, Additional file 3C), the predictors of subsequent relapse were psychiatric hospitalization in the year prior to study enrollment, earlier age of illness onset, and poorer level of functioning.

## Discussion

Although prior relapse has long been known to predict future relapse in the study of schizophrenia, this study provides new and useful information about the cost of relapse and its cost components in the United States, the predictors of relapse, and the important role of previous relapse, above and beyond information about patients' functioning and symptom levels. Current findings demonstrate that the annual mental health cost of relapsed patients is about 2 to 5 times higher than for non-relapsed patients, depending on whether the patients had relapsed in the 6 months prior to the 1-year study period. Prior relapse was found to be a strong predictor of subsequent relapse (overall accuracy 79%), showing that most patients who did not relapse in the 1-year study period (88%) were correctly identified as non-relapsed based on their previous 6-month non-relapse status (high specificity). Moreover, when assessing the costs of patients who relapsed during the 1-year period, those with prior relapse were about 2.8 times more costly. The cost differential was primarily driven by a higher number of hospitalizations and by longer hospital stay per admission. Importantly, the expected higher acute care costs of relapsed patients were accompanied by higher costs for various outpatient services and medication, suggesting that the cost of relapse is not confined to the cost of hospitalizations and emergency services as payers tend to believe, as relapse is also linked to more intense and thus more costly medication management, day treatment, individual therapy, and ACT/case management.

Consistent with prior research [1-3,6,9,21,22], the current analysis also found relapsed patients to have a more complex illness profile, which is not only associated with more severe symptomatology but also substance use, legal involvement, lower level of functioning, and poorer medication adherence. Furthermore, this study identified a small set of variables that help predict subsequent relapse in the usual treatment of schizophrenia, demonstrating the predictive value of prior relapse as a robust marker, along with prior medication nonadherence, younger age at illness onset, having health insurance, and poorer level of functioning. The use of these predictors in clinical practice may help improve allocation of resources, such as active case management and adherence interventions, since these programs aim to prevent relapse and hospitalization.

Current findings may also be of value for modeling the cost-effectiveness of treatment for schizophrenia and may also be of interest to payers and other health care decision makers, especially those involved in developing Medicare capitation models for patients with chronic conditions such as schizophrenia. Using a robust and simple clinical marker such as recent relapse may help improve the accuracy of Medicare risk adjustment models. This information may also be applicable to risk adjustments of premiums under Medicare Part D plans because drug expenditures in the previous year generally had been found to be strongly predictive of current-year drug expenditures for individuals [23,24]. Policy analysts have suggested that this expenditure pattern between prior and current years should be reflected in risk-adjustment formulae [25], and specifically in Medicare Part D [26].

This study has a number of strengths, including the breadth of its clinical and economic measures and the diversity of the patient population across geographies and health care systems, suggesting high generalizability of the findings. The study also has limitations. First is the potential for selection bias. Although propensity score matching was used to adjust for potential selection bias, such methods cannot account for all potentially confounding factors (i.e., unmeasured variables). For example, patients who were hospitalized continuously during the 1-year study period might have contributed disproportionately to overall costs. Accordingly, an additional sensitivity analysis was performed in which 13 such patients were excluded; results were highly consistent with the original findings (e.g., total cost was 2.2 times higher for patients with versus without prior relapse rather than 2.8 times higher). This study also assessed the potential impact of excluding patients from the analysis due to their lacking complete resource utilization data. The excluded patients differed significantly from the included patients on variables shown to be associated with relapse (e.g., younger age, prior hospitalizations, poorer adherence, and more severe symptoms), suggesting that the overall rate of relapse has likely been underestimated.

Second, the costs in this study only reflected direct mental health cost and not total health care costs because the US-SCAP study did not collect data on non-psychiatric resource utilization or indirect costs. Third, the study did not have complete mental health resources information for all patients across the 3-year study, thus curtailing the ability to assess change in costs over time. Fourth, the study did not assess the reason for patients' psychiatric hospitalization; thus there is a possibility that some hospitalizations may not have been directly linked to exacerbation of schizophrenia. And lastly, the results of this study may not be generalizable to patients with schizophrenia whose treatment is covered by private payers because public payers covered almost all US-SCAP participants [10,27].

## Conclusions

Relapse of patients with schizophrenia is associated with substantial direct mental health costs that extend beyond the cost of hospitalization to other costly outpatient services and medication costs. Findings highlight the economic impact of relapse and the importance of prior relapse as a predictor of subsequent relapse for clinicians and other health care decision makers. Future research is needed to evaluate the longer-term effects on patient outcomes and health care costs of targeting different interventions to patients at high risk of relapse.

## Competing interests

Dr. Ascher-Svanum is a full-time employee of Eli Lilly and Company. Drs. Zhu, Faries, Peng, and Conley are full-time employees of Lilly USA, LLC. All are shareholders in the study sponsor, Eli Lilly and Company. Dr. Salkever has served as a paid consultant to Eli Lilly and was an investigator on the US Schizophrenia Care and Assessment Program (US-SCAP). Dr. Slade served as a paid consultant to Eli Lilly on the US-SCAP, and his current work is supported in part by the US Department of Veterans Affairs, Capitol Network VISN5 Mental Illness Research and Education Clinical Center.

## Authors' contributions

HA-S conceived of the study, participated in its design, the analytical plan, the interpretation of the results, and helped write the manuscript. BZ performed the initial statistical analyses and participated in the design of the study and the analytical plan. DEF participated in the design of the study, the analytical plan, the interpretation of the results, and assisted in drafting the manuscript. DS and ES participated in the design of the study, the analytical plan, the interpretation of the results, and assisted in drafting the manuscript. They were also involved in preparing the resource utilization costing data of US-SCAP. XP performed the expanded statistical analyses, participated in the design of the study, the analytical plan, and the interpretation of the results. RRC assisted with the interpretation of the results and helped draft the manuscript. All authors read and approved the final manuscript.

## Acknowledgements

The US-SCAP study and its report were supported by Eli Lilly and Company, Indianapolis, IN, USA and administered by the Medstat Group. We wish to thank the site investigators and others who collaborated in the US-SCAP study: **Barrio C, Ph.D**., Center for Research on Child and Adolescent Mental Health Services, San Diego, CA; **Dunn LA, M.D**., Duke University Medical Center Department of Psychiatry, Durham, NC; **Gallucci G, M.D**., (previously) Johns Hopkins Bayview Medical Center and the University of Maryland Medical Systems, Baltimore, MD; **Garcia P, Ph.D**., Center for Research on Child and Adolescent Mental Health Services, San Diego, CA; **Harding C, Ph.D**., Boston University and Community Mental Health Centers in Denver, CO; **Hoff R, Ph.D., M.P.H**., West Haven Veterans Administration Medical Center (VAMC) and the Connecticut Mental Health Center (CMHC), West Haven, CT; **Hough R, Ph.D**., Center for Research on Child and Adolescent Mental Health Services, California, San Diego, CA; **Lehman AF, M.D**., Johns Hopkins Bayview Medical Center and the University of Maryland Medical Systems, Baltimore, MD; **Palmer L, Ph.D**., The Medstat Group, Inc., Washington, DC; **Rosenheck RA, M.D**., West Haven Veterans Administration Medical Center (VAMC) and the Connecticut Mental Health Center (CMHC), West Haven, CT; **Russo P, Ph.D., M.S.W., R.N**., (previously) The Medstat Group, Inc., Washington, DC; **Salkever D, Ph.D**., (previously) Johns Hopkins University, Department of Health Policy and Management, Baltimore, MD; **Saunders T, M.S**., Drug Abuse and Mental Health Program Office of District 7 and University of South Florida's Florida Mental Health Institute, Orlando, FL; **Shern D, Ph.D**., (previously) Drug Abuse and Mental Health Program Office of District 7 and University of South Florida's Florida Mental Health Institute, Orlando, FL; **Shumway M, Ph.D**., University of California at San Francisco, Department of Psychiatry, San Francisco, CA; **Slade E, Ph.D**., (previously) Johns Hopkins University, Department of Health Policy and Management, Baltimore, MD; **Swanson J, Ph.D**., Duke University Medical Center Department of Psychiatry, Durham, NC; **Swartz M, M.D**., Duke University Medical Center, Department of Psychiatry, Durham, NC.

## Pre-publication history

The pre-publication history for this paper can be accessed here:

http://www.biomedcentral.com/1471-244X/10/2/prepub

## Supplementary Material

Additional file 1 **Table S1. Baseline characteristics, direct annual mental health costs and cost components (in 2000 US dollars) for all 1,557 participants and for participants with and without prior relapse^a^**. Baseline sociodemographic and clinical characteristics, direct total annual mental health costs and cost components (in 2000 US dollars) for all 1,557 participants and for participants with and without prior relapse. Click here for file

Additional file 2 **Table S2. Baseline characteristics, total annual mental health costs, and cost components (in 2000 US dollars) by relapse status^†^**. Baseline sociodemographic and clinical characteristics, direct total annual mental health costs and cost components (in 2000 US dollars) for 4 groups that differed on relapse status prior to baseline. Click here for file

Additional file 3 **Table S3. Logistic regression analyses of relapse predictors for the 1,557 participants and by relapse status^a^**. Logistic regression analyses of relapse predictors for all the 1,557 participants, for Group RN versus RR (n = 310) and for Group NN versus NR (n = 1,247). Click here for file
