# Supplementary material for: The cost of relapse and the predictors of relapse in the treatment of schizophrenia
Source: BMC Psychiatry. 2010 Jan 7;10:2. doi: 10.1186/1471-244X-10-2 (PMC2817695; doi:10.1186/1471-244X-10-2)
Supplement: Additional file 1 — Table S1. Baseline characteristics, direct annual mental health costs and cost components (in 2000 US dollars) for all 1,557 participants and for participants with and without prior relapsea. Baseline sociodemographic and clinical characteristics, direct total annual mental health costs and cost components (in 2000 US dollars) for all 1,557 participants and for participants with and without prior relapse. [file 1471-244X-10-2-S1.DOC]

| **Table 1**  Baseline characteristics, direct annual mental health costs and cost components (in 2000 US dollars) for all 1,557 participants and for participants with and without prior relapse^a^ | | | | | | | | |  |
| --- | --- | --- | --- | --- | --- | --- | --- | --- | --- |
| Characteristic/ Cost parameter | All  (n=1,557) | |  | With Prior Relapse  (n=310) | |  | Without Prior Relapse  (n=1,247) | |  |
|  | N | % |  | N | % |  | N | % |  |
| Age (M ± SD, years)* | 42.4 ± 11.1 | |  | 39.3 ± 10.6 | |  | 43.1 ± 11.1 | |  |
| Age at onset of illness (M ± SD, years)* | 20.7 ± 8.9 | |  | 19.6 ± 8.5 | |  | 20.9 ± 9.0 | |  |
| Male | 948 | 60 |  | 178 | 57 |  | 770 | 61 |  |
| Single marital status | 938 | 60 |  | 189 | 61 |  | 749 | 60 |  |
| Race/ethnicity |  |  |  |  |  |  |  |  |  |
| Caucasian | 762 | 48 |  | 150 | 48 |  | 612 | 49 |  |
| African-American | 589 | 37 |  | 119 | 38 |  | 470 | 37 |  |
| Other | 66 | 4 |  | 17 | 5 |  | 49 | 3 |  |
| No health insurance | 104 | 6 |  | 27 | 8 |  | 77 | 6 |  |
| High school education or less | 1,047 | 67 |  | 207 | 67 |  | 840 | 68 |  |
| PANSS total score (M ± SD)* | 69.1 ± 18.4 | |  | 72.7 ± 18.4 | |  | 68.2 ± 18.2 | |  |
| MADRS score (M ± SD)* | 13.6 ± 10.2 | |  | 16.0 ± 11.2 | |  | 13.0 ± 9.8 | |  |
| Hospitalized in the 1 year before enrollment*^b^ | 714 | 45 |  | 294 | 94 |  | 420 | 33 |  |
| Comorbid substance use disorder* | 413 | 26 |  | 110 | 35 |  | 303 | 24 |  |
| Arrested* | 80 | 5 |  | 30 | 9 |  | 50 | 4 |  |
| Victimized* | 149 | 9 |  | 40 | 13 |  | 109 | 8 |  |
| Violent behaviors | 99 | 6 |  | 25 | 8 |  | 74 | 6 |  |
| Mental composite score per SF-12 (M ± SD)*** | 42.5 ± 12.9 | |  | 40.0 ± 13.4 | |  | 43.0 ± 12.8 | |  |
| Physical composite score per SF-12 (M ± SD) | 45.3 ± 13.1 | |  | 46.5 ± 12.9 | |  | 45.1 ± 13.2 | |  |
| Adherent with medication, per patient self-report*** | 1,407 | 92 |  | 261 | 86 |  | 1,146 | 94 |  |
| Medication Possession Ratio (MPR), (M ± SD)*** | 90.0 ± 25.3 | |  | 84.0 ± 30.2 | |  | 92.0 ± 23.7 | |  |
| Total annual cost** | 16,100±24,791 | |  | 33,297± 47,625 | |  | 11,825±10,638 | |  |
| Medications cost* | 4,819±3,858 | |  | 5,647±3,810 | |  | 4,613±3,844 | |  |
| Psychiatric hospitalizations cost** | 4,687±23,536 | |  | 19,298±48,545 | |  | 1,055±6,404 | |  |
| Day treatment cost** | 1,571±3,734 | |  | 1,816±3,968 | |  | 1,510±3,672 | |  |
| Emergency services cost** | 84±196 | |  | 166±271 | |  | 64±167 | |  |
| Psychosocial group therapy cost | 1,478±3,126 | |  | 1,541±3,060 | |  | 1,463±3,143 | |  |
| Medication management cost** | 1,187±1,331 | |  | 1,436±1,666 | |  | 1,125±1,227 | |  |
| Outpatient individual therapy cost** | 1,267±1,826 | |  | 1,987±2,555 | |  | 1,088±1,544 | |  |
| ACT/case management cost** | 1,006±958 | |  | 1,406±987 | |  | 907±925 | |  |

^a^Prior relapse, relapsed in the 6 months prior to the 1-year study period.

^b^Hospitalization in the 1 year prior to enrollment” includes hospitalization in months 7-12 prior to enrollment; thus participants without relapse in the prior 6 months could have been hospitalized in months 7-12 prior to enrollment.

MADRS, Montgomery-Åsberg Depression Rating Scale; PANSS, Positive and Negative Syndrome Scale; SF-12, 12-Item Short Form Health Survey. For the 10-item MADRS, possible scores range from 1 to 6, with higher scores indicating greater severity. For the 30-item PANSS assessment, possible scores range from 1 to 7, with higher scores indicating more severe symptoms. For the 12-item SF-12, possible scores range from 0 to 100 with higher standardized scores indicating better functioning.

P-values for comparisons of participants with versus without prior relapse:

* p<.05

** p<.01

*** p<.001
