# Supplementary material for: The cost of relapse and the predictors of relapse in the treatment of schizophrenia
Source: BMC Psychiatry. 2010 Jan 7;10:2. doi: 10.1186/1471-244X-10-2 (PMC2817695; doi:10.1186/1471-244X-10-2)
Supplement: Additional file 3 — Table S3. Logistic regression analyses of relapse predictors for the 1,557 participants and by relapse statusa. Logistic regression analyses of relapse predictors for all the 1,557 participants, for Group RN versus RR (n = 310) and for Group NN versus NR (n = 1,247). [file 1471-244X-10-2-S3.DOC]

| **Table 3**  Logistic regression analyses of relapse predictors for the 1,557 participants and by relapse status^a^ | | | |
| --- | --- | --- | --- |
|  | | | |
| **Outcomes and variables** | | | |
| **A. All patients (n=1557)** | | | |
|  | OR | 95% CI | p-value |
| Nonadherent vs. adherent with medication | 1.79 | 1.04–3.06 | .034 |
| Having health insurance vs. not | 2.32 | 1.11–4.85 | .025 |
| Prior relapse vs. not | 4.23 | 2.41–7.44 | <.001 |
| Number of prior hospital admissions | 1.45 | .98–2.15 | .065 |
| Age at illness onset | .97 | .96–.99 | .005 |
| SF-12 Physical Composite Score | .98 | .97–.99 | .004 |
| SF-12 Mental Composite Score | .98 | .97–.99 | .001 |
| Comments: A 10 units lower score on the SF-12 Physical Composite Score and Mental Composite Score are associated with 18% and 16% increase respectively in the predicted odds of a psychiatric hospitalization. Similarly, a year later illness onset and 1 additional prior psychiatric hospitalization are associated with 3% decrease and 45% increase respectively in the odds of a psychiatric hospitalization prediction. | | | |
| **B. Group RN vs. RR (n=310)** | | | |
|  |  |  |  |
| PANSS total score | 1.02 | 1.01–1.03 | .009 |
| Number of prior hospital admissions | 1.63 | 1.12–2.37 | .011 |
| Comments: A 10 units increase in PANSS total score and 1 additional prior psychiatric hospitalization are associated with 22% and 63% increase in the predicted odds of a psychiatric hospitalization | | | |
| **C. Group NN vs. NR (n=1247)** | | | |
|  |  |  |  |
| Nonadherent vs. adherent with medication | 1.30 | .59–2.85 | .517 |
| Age at illness onset | .97 | .94–.99 | .003 |
| PANSS total score | 1.00 | .99–1.01 | .573 |
| SF-12 Mental Composite Score | .98 | .96–.99 | .004 |
| SF-12 Physical Composite Score | .97 | .95–.98 | <.001 |
| Hospitalized in the 1 year prior to enrollment | 5.85 | 3.82–8.93 | <.001 |
| Comments: A 10 units lower score on SF-12 Physical Composite Score and Mental Composite Score are associated with 18% and 26% increase in the predicted odds of a psychiatric hospitalization. Similarly, 1 year later illness onset is associated with 3% decrease in the predicted odds of a psychiatric hospitalization prediction. | | | |
| ^a^The first letter (either N=no relapse or R=relapse) represents prior relapse status. The second letter (either N=no relapse or R=relapse) represents relapse status in the 1-year study period (e.g., NR means not relapsed in the prior 6 months and relapsed in the subsequent 1-year study period).  Covariates: age, gender, race (White, Black), age at illness onset, single marital status, high school education or less, schizoaffective disorder, having health insurance, substance use, arrest, victimization, violent, MADRS score, Positive and Negative Syndrome Scale (PANSS) total, positive, negative, and general psychopathology scores, at least moderately ill (PANSS≥75), inpatient at enrollment in US-SCAP study; relapsed, Medication Possession Ratio (MPR), number of psychiatric hospitalizations, any psychiatric hospitalization, any use of crisis bed, any use of emergency services (all in the 6 months prior to 1-year study period); suicide attempt, adherent with medication (in previous 4 weeks); physical and mental composite scores per 12-Item Short Form Health Survey (SF-12) with lower SF-12 composite scores reflecting standardized scores <50. Stepwise selection was utilized to build each prediction model. The final models may have nonsignificant predictors due to other predictors added to the model afterwards. | | | |
